# A Fast Slicing Method for Colored Models Based on Colored Triangular Prism and OpenGL

**DOI:** 10.3390/mi16020199

**Published:** 2025-02-09

**Authors:** Lei Xia, Ran Yan

**Affiliations:** College of Mechanical Engineering, Chongqing University of Technology, Chongqing 400054, China

**Keywords:** colored 3D printing, colored triangular prism, OpenGL, model slicing

## Abstract

Colored 3D printing, as one of the crucial directions in 3D printing technology, has been widely applied in various fields in recent years. Compared to traditional 3D printing, colored 3D printing introduces color information to achieve multi-material identification of different regions in the model structure, enabling the fabrication of heterogeneous and complex components. This presents unique advantages in both visual effects and functionality, making it of significant value in fields such as metal manufacturing, bioengineering, and artistic design. However, during the construction of colored models, technical challenges such as low-slicing contour accuracy and poor color reproduction persist. Existing slicing methods for colored models are often accompanied by contour offset, deformation, color distortion, and low rendering efficiency, severely limiting the application scope of colored 3D printing technology. To address these challenges, this paper proposes a “Fast Slicing Method for Colored Models Based on Colored Triangular Prisms and OpenGL”. This method first constructs colored triangular prisms to effectively solve the problems of color contour offset and deformation, achieving uniform thickness offset of the colors. Then, by utilizing OpenGL rendering technology, the method overcomes color abruptness, simplifies bitmap rendering processes, and ensures smooth color transitions while significantly improving rendering efficiency. In summary, the proposed slicing method can effectively enhance the accuracy of slicing contours and color reproduction, significantly expanding the application range of colored 3D printing.

## 1. Introduction

Colored 3D printing, as an important direction in 3D printing technology, has found widespread applications in various fields in recent years. The technology originates from traditional paper-based color inkjet printing combined with binder jetting 3D printing, where a printhead selectively deposits binder onto a powder bed to form solid structures. By replacing single-color ink with cyan, magenta, yellow, and black (CMYK) inks, nearly all colors can be reproduced through precise mixing [1]. Compared to traditional monochrome printing, colored 3D printing offers unique advantages in both visual effects and functionality by introducing color information. It allows for identifying multiple materials in different regions of the 3D printed structure, enabling the fabrication of heterogeneous and complex components. For instance, mixing different metal or ceramic powders for powder-based printing can meet special requirements [2,3,4]. In biomedical fields, colored 3D printing can precisely express different types of tissues through multi-colored materials, intuitively simulating human structures and improving the accuracy of diagnosis and treatment plans. For example, in cases where tumors have invaded adjacent tissues, preoperative imaging techniques such as CT and MRI provide only 2D visualizations, making it difficult for surgeons to determine the optimal incision points. However, by 3D printing a full-color model of the tumor—complete with bone, muscle, arteries, and veins—surgeons can physically practice the procedure beforehand, dramatically improving surgical success rates [5]. At the same time, multi-material and multi-color printing technologies allow for precise control of cell distribution and layer structures, promoting the construction of complex biological tissues [6,7,8,9]. Additionally, multi-material and multi-nozzle inkjet printing enables the fabrication of high-precision voxelated soft matter, allowing precise control over individual voxels to create intricate material distributions [10]. Overall, colored 3D printing technology offers higher design freedom and precision across various industries, particularly biomedical and jewelry design. It improves diagnostic accuracy and drives personalized customization and production efficiency, demonstrating significant practical value and broad development prospects [11,12,13].

**Figure 1:**
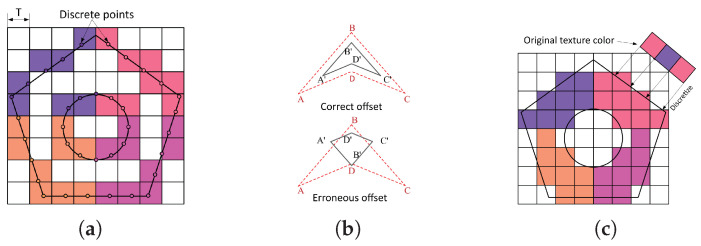
(**a**) Color contour lines with varying point sizes. (**b**) Contour offset deformation. (**c**) Mismatch between texture and slicing contour.

Compared to traditional 3D printing models, colored models introduce an additional dimension of color information, making the slicing process more complex. It is necessary to define regions of different colors on the surface, consider depth, and account for color transitions. However, existing methods for processing colored models mainly face two key challenges: slicing contour accuracy and color reproduction. Color-based slicing methods, which exploit the model’s continuity, have advantages such as low computational complexity but cannot generate color thickness [14]. To address this, Xu et al. [15] proposed a slicing method based on vertex color, which implements color gradients by interpolating the endpoints of contour lines, calculating the coordinates and color information of interpolation points for each segment, and replacing the color contour lines with points of varying sizes (*T*, half the thickness) (Figure 1a). However, it cannot restore true color information or accurate slicing contours. Tian et al. [16] expressed color texture thickness through contour offset, but this process tends to introduce deformation, affecting contour accuracy. Specifically, in Figure 1b, the polygon A,B,C,D (red dashed line) represents the vertices of the contour before offsetting, while the deformed contour after offsetting is shown by the vertices $A^{\prime },B^{\prime },C^{\prime },D^{\prime }$ (black solid line). This deformation is caused by geometric distortion during the offset process, leading to the sliced contour failing to accurately reflect the original model’s shape. A point-based coloring method using triangular prisms [17] ensures contour accuracy (Figure 1c). However, the computational complexity during scanline filling is high, and when discretizing the original texture color into a bitmap, the size matching relationship is not considered, leading to a direct mapping that limits the color restoration accuracy.

To address these issues, this paper proposes a “Fast Slicing Method for Colored Models Based on Colored Triangular Prisms and OpenGL” based on the incremental approach. First, the model structure and surface color information are analyzed. Equal-thickness color regions are created on the model’s surface, and a fast slicing algorithm is used to slice the model contours. Then, based on the slicing results, color information is calculated and passed to OpenGL for bitmap processing. This enables the precise calculation of color values for each pixel in the equal-thickness color regions. This method significantly improves slicing efficiency while optimizing color expression and texture detail reproduction, effectively solving the problems of slicing contour accuracy and color reproduction.

## 2. Materials and Methods

### 2.1. Construction of Colored Triangular Prisms and Color Interpolation Calculation

Translating the triangle along its normal direction achieves the color triangular prism offset. Together with the original triangle vertices, it forms the triangular prism space. All triangles on the model’s surface are transformed into colored triangular prisms, and the collection of these colored triangular prisms forms the required equal-thickness color regions.

Let the three vertices of a triangular face be $v_{1}$, $v_{2}$, and $v_{3}$, with corresponding color space coordinates $c_{1}$, $c_{2}$, and $c_{3}$. The color information at an arbitrary point (*p*) in the triangular prism is given by the coordinates in the color coordinate system corresponding to the foot of the regular line.

According to the barycentric interpolation principle, for any point (*v*) inside the triangle, with known coordinates $v_{1}$, $v_{2}$, and $v_{3}$, there exist scalar weights *s* and *t*, such that

$$v=\left(1-s-t\right)v_{1}+sv_{2}+tv_{3}.$$

In the slicing plane, introducing the standard depth *l* and letting the unit normal vector of the triangular face be *N*, the coordinates of any point (*p*) in the triangular prism in the coordinate system are given by the following:

$$\begin{array}{ccc} p & = & v+lN\\ & = & \left[\begin{array}{cc} x_{2}-x_{1} & x_{3}-x_{1}\\ y_{2}-y_{1} & y_{3}-y_{1}\\ z_{2}-z_{1} & z_{3}-z_{1} \end{array}\right]\left[\begin{array}{c} s\\ t \end{array}\right]+\left[\begin{array}{c} x_{1}\\ y_{1}\\ z_{1} \end{array}\right]+l\left[\begin{array}{c} -x_{n}\\ -y_{n}\\ -z_{n} \end{array}\right]\\ & = & \left[\begin{array}{ccc} x_{2}-x_{1} & x_{3}-x_{1} & -x_{n}\\ y_{2}-y_{1} & y_{3}-y_{1} & -y_{n}\\ z_{2}-z_{1} & z_{3}-z_{1} & -z_{n} \end{array}\right]\left[\begin{array}{c} s\\ t\\ l \end{array}\right]+\left[\begin{array}{c} x_{1}\\ y_{1}\\ z_{1} \end{array}\right] \end{array}$$

By introducing a color information coordinate system vector *C*, and recognizing that the vectors formed by the triangle’s sides and its unit normal vector are linearly independent, we can invert the system and calculate the color information coordinates at any point in the color triangular prism coordinate system *D*:

(1) $$\left\{\begin{array}{c} D=C\left[\begin{array}{c} s\\ t\\ l \end{array}\right]+C_{1}=CX^{-1}\left[\begin{array}{c} x-x_{1}\\ y-y_{1}\\ z-z_{1} \end{array}\right]+C_{1}\\ X=\left[\begin{array}{ccc} x_{2}-x_{1} & x_{3}-x_{1} & -x_{n}\\ y_{2}-y_{1} & y_{3}-y_{1} & -y_{n}\\ z_{2}-z_{1} & z_{3}-z_{1} & -z_{n} \end{array}\right] \end{array}\right.$$

### 2.2. OpenGL-Based Out-of-Bounds and Overlap Color Sampling Method

In colored 3D printing, accurately and efficiently acquiring the color information of the model’s slices is key to achieving precise printing. As an incremental computation approach, the point-by-point method significantly improves efficiency by reducing repetitive calculations. However, it requires point-by-point computation, and managing and traversing active edge tables can be cumbersome. Based on the point-by-point method, this paper combines OpenGL rendering techniques to avoid the complicated processes of handling out-of-bounds and overlapping color sampling, thus optimizing the color triangular prism offset algorithm. This makes the color interpolation calculations more precise and efficient.

**Figure 2:**
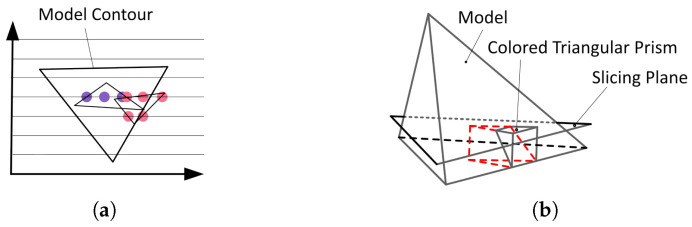
(**a**) Point-by-point color sampling. (**b**) Triangular prism out-of-bounds.

The point-by-point method calculates each pixel (Figure 2a) sequentially with fixed increments. For each scanline, based on Equation (1), the value of the first point is calculated directly using matrix multiplication, and subsequent points are updated by adding the increment to the previous point. This simplifies multiple multiplication and addition operations into just a few additions, significantly improving computational efficiency. Additionally, since triangular prisms may go out of bounds or overlap (Figure 2b), their coordinates must be mapped to the bitmap coordinate system. Because bitmap coordinates need to be rounded, some coordinates may become misaligned, resulting in artifacts such as jagged edges or color discontinuities. Out-of-bounds and overlapping regions require real-time calculations and updates via the active edge table, making the process complex.

This paper utilizes OpenGL rendering for the bitmap to optimize this process, employing depth and stencil tests to resolve the out-of-bounds and overlap color sampling issues. Only the relevant information for each vertex is calculated during the computation, reducing computational load. There is no need to focus on each triangular prism’s out-of-bounds or overlapping status. Instead, OpenGL configuration is performed only when generating the final bitmap, significantly lowering the algorithm’s complexity.

### 2.3. Steps for the Fast Slicing of Colored 3D Models Based on OpenGL

Based on the above triangular prism offset, constrained Delaunay triangulation algorithm, and OpenGL bitmap rendering, let the surface colored model be *M*, with the thickness of the equal-thickness color regions as *d*, and bitmap resolution as Dpi.

The model *M* is sliced from low to high with a thickness of ▵z, and the corresponding colored bitmap and contour slices for each layer of *M* are obtained from low to high. The overall steps are as follows:

1. Read the 3MF file of model *M*. Extract the color information of its triangular faces. For each triangular face, construct the colored triangular prism based on the thickness dd, calculate the constant matrices $A=CX^{-1}$ and $B=C_{1}-CX^{-1}$, and sort the triangular prisms by the z-coordinate in ascending order. At the same time, reconstruct the face-edge topology based on the shared vertex topology of the triangular faces of *M*.
2. For the current z-value, quickly find the set of triangular prisms of $T_{z}$ to be sliced. Use the fast slicing algorithm to slice both *M* and $T_{z}$. Set the slicing depth of the model’s contour (*L*) to 0. Calculate the colored triangular prism contour slices based on the color type, obtaining the triangular prism contour slice $L_{t}$, and compute the color and depth for all its vertices.
3. Perform Delaunay triangulation on *L* with constraints to obtain the triangulated vertex indices *I*, and simultaneously triangulate $L_{t}$. Calculate and set the scaling factor scale factor based on Dpi, and set the view matrix to point from the X–Y plane to the negative Z-axis. OpenGL is used in orthogonal projection mode, enabling depth and stencil testing. Clear the depth buffer and stencil buffer in OpenGL and set the stencil test to render pixels with a stencil value of 255. Set the output color to the material color of *M*, then pass the vertices of *L* into OpenGL and render them in the order specified by *I*. Set the stencil test to render only pixels with a value of 255, enable texture sampling, and input $L_{t}$ into OpenGL for rendering. Complete the bitmap conversion and obtain the colored slice bitmap.

### 2.4. Experimental Verification

The experimental 3D printing device is a self-built 3D printer with an Epson DX-5 F18600 high-precision color printhead, which is sourced from Seiko Epson Corporation, Nagano, Japan. It has an inherent horizontal resolution of 1440 dpi, with the printhead containing 1440 nozzles distributed in 8 rows (180 nozzles per row) and controlled in CMYK color mode (cyan, magenta, yellow, and black). The device uses a self-developed printhead control card. The printing parameters are a roller speed of 10 r/min, powder spreading speed of 30 mm/s, and printing speed of 20 mm/s. The printing material is calcium sulfate-based powder mixed with ceramic composite ingredients to enhance mechanical strength and surface quality. This material offers excellent printing adaptability, ensuring precise model contours and accurate color reproduction, providing a reliable basis for experimental validation.

## 3. Results and Discussion

### 3.1. Construction of Colored Triangular Prisms and Color Sampling Strategy

**Figure 3:**
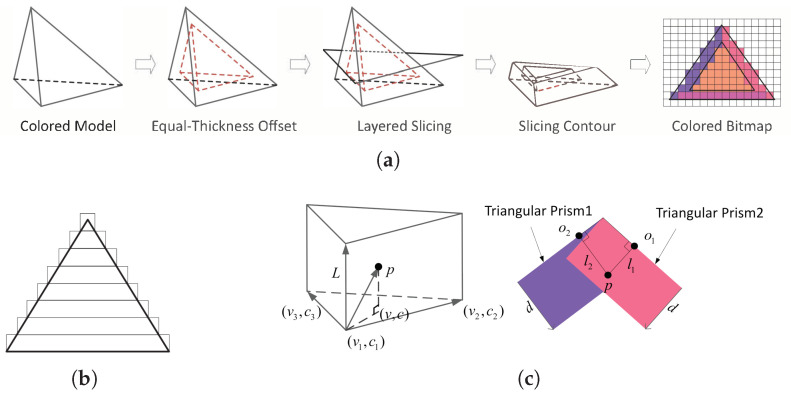
(**a**) Colored 3D model slicing process. (**b**) Out-of-bounds. (**c**) Construction of colored triangular prisms and overlap color sampling.

In slicing surface-colored 3D printing models with equal-thickness slices (Figure 3a), the color thickness on the model’s surface remains consistent, and an “equal-thickness offset” is required to ensure the accuracy of the printed shape. This process is based on the triangular faces of the model’s surface. By offsetting along the inward normal direction of each triangle, new triangle positions are generated. The offset and original points form triangular prisms, thus achieving an equal-thickness offset. This operation is performed on all triangles, resulting in a set of triangular prisms representing the model’s color information.

Additionally, the constructed colored triangular prisms may extend beyond the current layer’s contour (Figure 3b). In this case, the out-of-bounds triangular prisms need to be handled separately to ensure the completeness of the model’s boundary and the accuracy of the color. A distance-based color selection strategy is used when a point lies within the range of multiple triangular prisms. The perpendicular distance from the point to each triangular prism’s base (model triangle face) is computed. The color of the nearest triangular prism is selected as the point’s final color (Figure 3c). This effectively avoids color conflicts when multiple triangular prisms overlap, ensuring that the color of each point matches the nearest triangular prism.

### 3.2. Efficient OpenGL-Based Out-of-Bounds and Overlap Color Sampling Method

**Figure 4:**
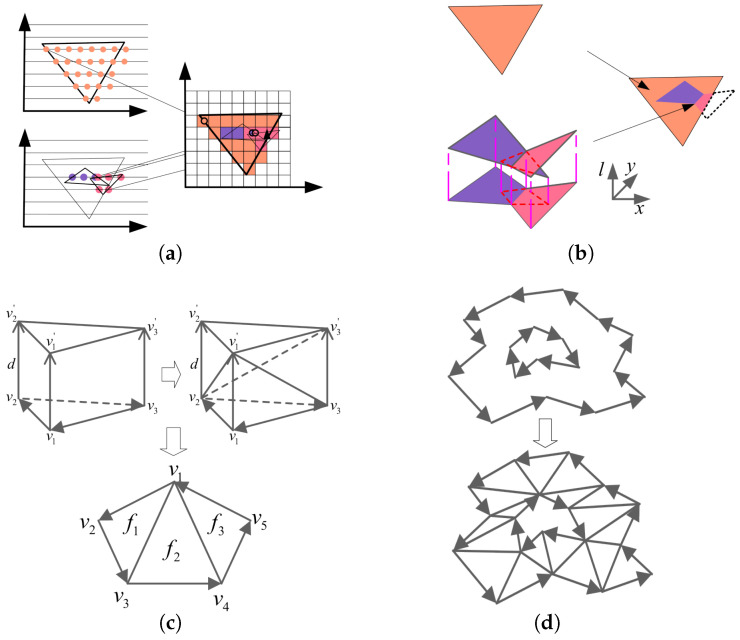
(**a**) Point-by-point coloring calculation. (**b**) OpenGL coloring calculation. (**c**) Triangular triangulation of triangular prisms and slices. (**d**) Triangular triangulation of contour slices.

In the point-by-point method, each coloring point requires multiple queries and calculations (Figure 4a). To improve computational efficiency, the point-by-point method sorts the triangular prism and model contours. The method optimizes specific calculations by progressively querying whether the current point lies within the contour and whether it is closer to the corresponding contour. However, its complexity remains high, and the computational load increases quadratically with the height and the number of triangular prisms, following $O(n^{2})$, requiring recalculation at different resolutions (Dpi), and further increasing complexity and computational burden. Additionally, this discretized color sampling method cannot guarantee smooth color transitions between adjacent points, leading to aliasing distortion.

In contrast, the OpenGL-based method significantly simplifies the calculation process by computing only the information of each slice vertex without focusing on the contours’ overlap relationships. When generating the rendering bitmap, it is directly created according to the resolution, greatly reducing computational load and lowering algorithmic complexity (Figure 4b).

Optimization of Depth Testing: For each slicing plane, all slice vertices share the same z-coordinate. Therefore, these vertices’ z-values are replaced by the corresponding depth values of the triangular prisms. A depth value is maintained for each triangular vertex to represent its distance from the observer’s viewpoint. During rendering, the depth buffer is initialized to the maximum value. OpenGL updates the pixel’s color and depth value when the depth value of a portion of the geometry is less than the current value in the depth buffer (i.e., that part is closer to the observer). Otherwise, the rendering engine discards the rendering portion, avoiding complex active edge table calculations and queries, significantly improving rendering efficiency.

Optimization of Stencil Testing: Using stencil testing, the correct contour region of the model is defined in the stencil buffer before rendering, and pixel rendering is controlled based on preset rules. During the actual rendering of the colored bitmap, pixels are only allowed to be rendered if they lie within the predefined contour. This avoids rendering the regions outside the contours, reducing the subsequent depth and color calculation overhead.

Since the fast slicing algorithm only supports triangular faces and cannot directly handle complex polygon contours, and since the slicing results are typically irregular complex polygons, the complex polygon contours need to be split into multiple non-overlapping triangles before being passed to OpenGL for rendering. These triangles can fully cover the original shape. Through triangulation, irregular complex polygons can be converted into triangles suitable for processing by the fast slicing algorithm, ensuring that OpenGL rendering can efficiently utilize the GPU’s parallel processing capabilities.

Triangulation of triangular prisms focuses on their three rectangular faces. The triangular subdivision of rectangular faces is relatively simple. This paper adopts the diagonal subdivision (Figure 4c): each rectangle is divided into two triangles by a pair of diagonals. The vertex sequence after subdivision is fixed. In three-dimensional space, any section produced by the intersection of a plane and a convex polyhedron will be convex. Since a convex polygon is a simple polygon, any line segment between two points will lie entirely inside the polygon. This convex property ensures no concave or hole regions in the polygon, allowing it to be directly triangulated using the polygon’s vertices in order without complex preprocessing.

Triangulation of arbitrary 2D polygons is more complex, but related technologies are well-developed, and many open-source libraries are available. In this paper, we use the compact and efficient Triangle library written in C to perform constrained Delaunay triangulation (Figure 4d) on the simple polygons of the edge contours, obtaining correct contour triangulations.

### 3.3. Efficiency Improvement of the Fast Colored 3D Model Slicing Algorithm

In the experiment, a solid cube with dimensions 300 × 300 × 300 mm is used as the test object, initially displayed as white. The experiment was conducted on a platform equipped with an Intel Core i5 processor and GeForce RTX 2060 graphics card, using C++ programming and OpenGL and Qt for graphics rendering and interface interaction.

The incremental algorithm is selected as the benchmark to evaluate the proposed algorithm’s performance further. Using the scanline method, the incremental method quickly determines if a point lies within a triangular prism and reduces the computational load with incremental addition. The primary computational load of the algorithm focuses on processing coloring points, and the surface color layer thickness and slicing resolution influence the number of coloring points. Therefore, in this study, the time efficiency of the two algorithms in processing colored slices is compared using the resolution parameter, especially the performance of the algorithms at different resolutions.

**Figure 5:**
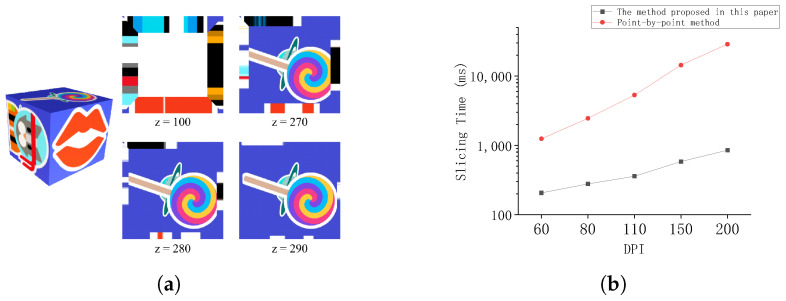
(**a**) Bitmap slices at different heights. (**b**) Algorithm runtime for 50mm thickness at different resolutions.

The color-slicing experiment results show that the proposed algorithm can effectively and accurately handle overlapping regions of multiple triangular prisms at a resolution of 60 dpi and a color layer thickness of 50 mm. In these overlapping regions, the boundaries of color transitions are visible, and the resulting color image of the slice demonstrates rich color depth while successfully avoiding color banding (Figure 5a). This verifies the superiority and efficiency of the proposed algorithm in handling complex overlapping regions.

Under the conditions of a slicing height of 200 mm and a thickness of 20 mm, experimental comparisons demonstrate, as shown in Figure 5a, that with increasing resolution, the proposed algorithm exhibits significant advantages in time efficiency compared to the incremental method. Additionally, the rate of time increase is noticeably lower than that of the incremental method. This indicates that the proposed algorithm performs more stably in high-resolution slicing tasks and is more efficient in handling complex colored slicing tasks.

**Figure 6:**
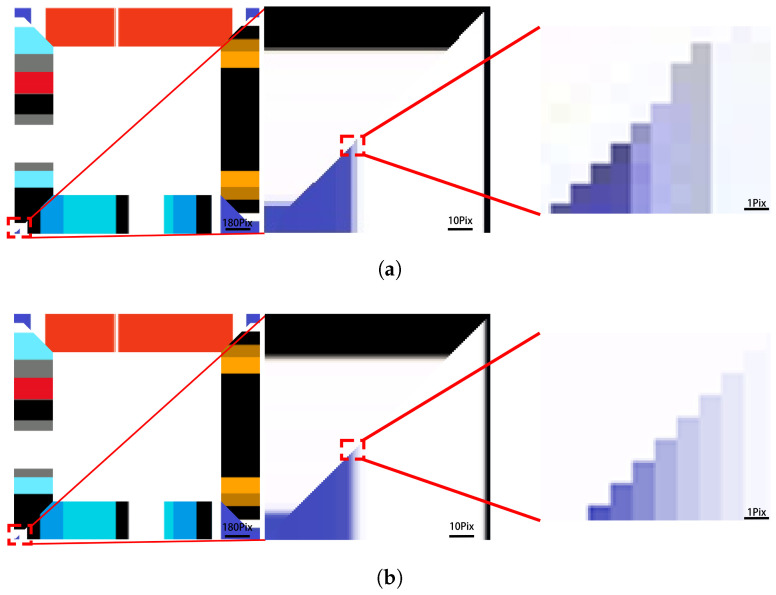
(**a**) Detail representation using the point-by-point method. (**b**) Detail representation using the proposed algorithm.

When the resolution is increased to 200 dpi, as shown in Figure 6b, the proposed algorithm performs better in anti-aliasing and processes color transition areas more finely, significantly improving the image’s visual effect. Compared to traditional methods that calculate color information point-by-point based on the incremental method, the OpenGL-based rendering approach treats triangle faces as the processing unit, improving coloring speed while ensuring more refined details, thus further enhancing overall rendering quality. This method’s efficiency and detailed representation make it more suitable for large-scale 3D printing applications.

### 3.4. Experimental Validation

The ceramic color printing device uses a single printhead scheme. The print area is relatively small to ensure processing efficiency, with a maximum printing area of 400 × 400 × 600 mm. The ceramic color printing device uses the Epson DX-5 F18600 high-precision color printhead and is equipped with a self-developed printhead control card. Due to the lower powder delivery scheme, all powder materials must be added to the powder supply tank before printing, with the auxiliary powder supply equipment separate from the device and not requiring upper-level control.

**Figure 7:**
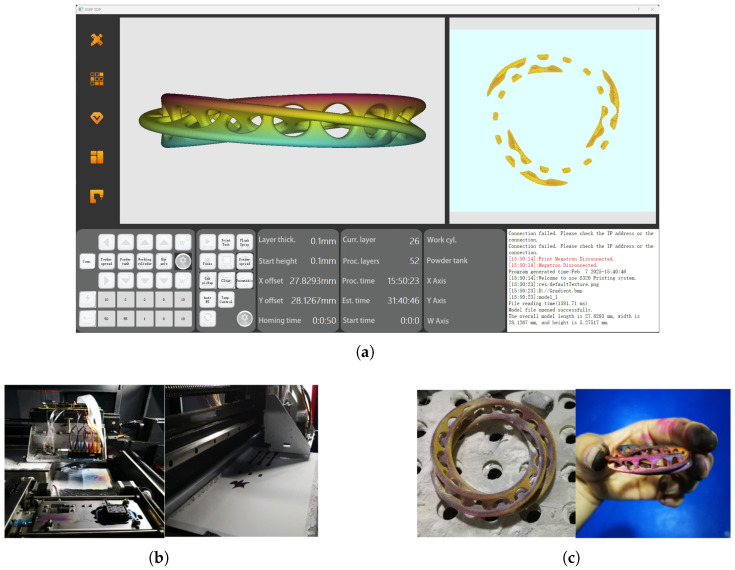
(**a**) BSRP 3D printing software. (**b**) Printing equipment and process. (**c**) Printed physical result.

The printing process was conducted using our self-developed BSRP 3D printing software (version 1.0.0.0.) (Figure 7a). The experiments were performed on the specified equipment using calcium sulfate-based powder mixed with ceramic composite materials to enhance the mechanical properties of the printed objects. As shown in Figure 7b, the printhead has an inherent resolution of 1440 DPI, with a total of 1440 nozzles, arranged in eight rows of 180 nozzles per row. These eight rows are grouped into four sets corresponding to CMYK inks. The printing parameters used include a powder spreading roller speed of 10 r/min, a powder spreading speed of 30 mm/s, and a printing speed of 20 mm/s.

After printing, the object is left to react and solidify to ensure structural stability and integrity, followed by manual cleaning to remove residual powder from the surface. The final printed object is shown in Figure 7c, demonstrating accurate color reproduction. In addition to the results presented, this printing system is capable of fabricating a wide variety of models and components, with specific designs tailored to user requirements.

## 4. Conclusions

In conclusion, colored 3D printing, as a technology with broad application prospects, faces limitations in development due to issues such as slicing contour accuracy and color reproduction. The “Fast Slicing Method for Colored Models Based on Colored Triangular Prisms and OpenGL” proposed in this paper effectively addresses the key challenges in traditional slicing methods, achieving high precision and efficiency in colored model slicing. This method not only enhances the technical performance of colored 3D printing but also provides new possibilities for its application in a broader range of fields, offering significant research and practical value.

## Data Availability

Due to privacy or ethical restrictions, the data supporting the reported results cannot be made available.
